# Relationship between salivary cortisol and depression in adolescent survivors of a major natural disaster

**DOI:** 10.1007/s12576-014-0315-x

**Published:** 2014-04-18

**Authors:** Takashi Yonekura, Kazunori Takeda, Vivek Shetty, Masaki Yamaguchi

**Affiliations:** 1 Biomedical Engineering and Robotics Laboratory, Graduate School of Engineering, Iwate University, 4-3-5 Ueda, Morioka, 020-8551 Japan; 2 Graduate Course of Disability Science, University of Tsukuba, Ibaraki 1-1-1 Tennodai, Tsukuba, Ibaraki 305-8577 Japan; 3 Section of Oral and Maxillofacial Surgery, UCLA School of Dentistry, University of California, Los Angeles, 10833 Le Conte Avenue, Los Angeles, CA 90095-1668 USA

**Keywords:** Cortisol, Depression, GHQ, Adolescents, Natural disasters

## Abstract

The purpose of this study was to determine the utility of salivary cortisol levels for screening mental states such as depression in adolescents following a natural disaster. We examined the relationship of salivary cortisol levels in adolescent survivors of the 2011 Tohoku Earthquake with the depression subscale of the 28-item General Health Questionnaire (GHQ). Subjects were 63 adolescent survivors (age = 14.29 years ± 0.51) who were administered the GHQ and provided saliva samples thrice daily (morning, afternoon and evening) over the course of 3 days. Based on the GHQ-depression subscores, subjects were divided into low and high depression groups. About 22 % of the subjects were classified into the high symptom group. When data collected over 3 days were used, a significant difference was observed between the two groups in the salivary cortisol levels at the evening time point as well the ratio of the morning/evening levels (*p* < 0.05). Analyzed by means of receiver-operating characteristic curves, the morning/evening ratios showed a good power in discriminating between subjects with and without depressive symptoms. Our study suggests that repeated measurement of salivary cortisol levels over 3 days has utility in screening for depressive states in adolescents following a natural disaster.

## Introduction

The Great East Japan Earthquake (the Tohoku Earthquake) of 2011 was one of the most devastating natural disasters ever to hit Japan [1]. Beyond the enormous loss of life and property, the extraordinary combination of a major earthquake, tsunami and radiation disaster provoked a lot of emotional and psychosocial turmoil in the survivors [2, 3]. As with most natural disasters, the initial negative psychological reactions usually resolve over the ensuing weeks and months [4]. However, studies of the mental consequences of natural disasters suggest that significant subsets of the survivors would continue to manifest debilitating stress disorders, including post-traumatic stress and depression, for prolonged periods of time [4, 5].

Children and adolescents tend to be the most vulnerable members of communities affected by natural disasters and are particularly prone to resulting mental disorders [6]. Developing in the weeks and months following the traumatic event, the severity of their psychological symptoms vary as a function of their level of exposure to the event, loss of loved ones, personal injury, level of parental support and sense of dislocation [7]. Once established, the mental disorders can produce a range of consequences including relationship difficulties, poor educational and vocational outcomes, recurrent illnesses and substance abuse. Although early identification and treatment of such disorders can lead to improved outcomes, many young people experiencing mental health problems do not seek help [8]. Thus, there is a need for a systematic post-disaster psychological assessment of children, and adolescents in particular, and the population in general [9–11]. Broad-based screening for adverse psychological repercussions of a natural disaster will help estimate community needs for mental health services, identify those at highest risk for mental health problems, and provide them with timely and appropriate interventions and needed support.

An essential element of large-scale psychological screenings in affected communities is the availability of convenient, valid and reliable assessment tools [11, 12]. To be useful and practical in a setting depleted of resources, infrastructure and trained personnel, these tools need to be inexpensive, brief, scalable and easily administered by non-specialists in a variety of settings. Typically, psychological distress questionnaires are used as screening instruments for mental disorders in clinical and epidemiologic settings. Some of the commonly used assessment instruments include the Trauma Screening Questionnaire, the Post-Traumatic Diagnostic Scale (PDS) and the General Health Questionnaire [4, 12–14]. However, the use of psychometric instruments does have limitations. Eliciting patients’ subjective descriptions of their symptoms introduces numerous reliability and validity issues, especially across cultures and ethnic groups [15, 16]. The psychometric properties of self-report measures also suffer from the fact that stress symptoms can be consciously or unconsciously minimized or exaggerated by the respondent [17]. Furthermore, the time-intensive administration of many psychological assessments can render them impractical in community settings with constrained resources [18].

To address the demand for field-practical adjuncts and practical psychological screening tools, our group has been developing salivary biosensors to provide point-of-care measurement of biomarkers reflective of the neuroendocrine response to stress. We posit that reliable, quantifiable and readily obtained biological measures that reflect existing psychological states could facilitate rapid and objective screening of post-traumatic psychopathology including depression. One focus of our efforts has been the measurement of salivary cortisol, a commonly used biomarker of the stress response and psychopathology [18–21]. Salivary cortisol has been used in a range of clinical studies of stress-related diseases including depression [15], PTSD [16] and irritable bowel syndrome [17]. Salivary cortisol correlates very well with free cortisol in the blood (*R* = 0.71–0.97) [18, 19], thus rendering salivary cortisol very attractive for non-invasive measurement of the neuroendocrine perturbations associated with traumatic stress reactions.

To determine the utility of salivary cortisol levels for screening mental states following a natural disaster, we examined the relationship of salivary cortisol levels in adolescent survivors of the Tohoku earthquake with a conventional psychometric scale for depression. Additionally, we explored the optimal timing and frequency of the salivary cortisol measurement in order to assess depressive symptoms.

## Methods

### Study design and setting

The study was conducted in Iwate Prefecture (Japan), one of the areas worst hit by the Tohoku earthquake, where the ensuing tsunami claimed 4,659 lives, with 1,633 people missing. The study was carried out in collaboration with the Iwate School District, and the study design and protocol were reviewed and approved by the Ethics Committee of Iwate University. We enrolled a convenience sample of 63 junior high-school students from the coastal regions of Iwate Prefecture. Study participation was voluntary, and the study protocol was fully explained to the subjects as well as their guardians (biological or adoptive parents). Informed consent was obtained from both.

**Fig. 1:**
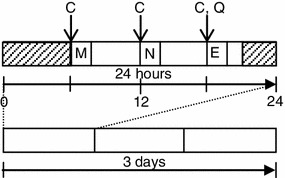
Protocol for collecting the saliva and subjective evaluation. *C* collection of the saliva, *Q* general health questionnaires, *M* morning, *N* noon, *E* evening

All enrolled subjects were given a batch of collection kits for collecting spit samples as well as self-report questionnaires for concomitant assessment of mental health states. The assessments were carried out in the subjects’ own homes over 3 consecutive days of a national holiday extending from Saturday 14 July to Monday 16 July 2012 (Fig. 1).

### Psychological assessment

Assessment of mental health states was performed with the Japanese version of the General Health Questionnaire-28 (GHQ-28) [22]. The GHQ is a widely used measure designed to assess current mental well-being. Developed as a screening tool to detect those likely to have or be at risk of developing psychiatric disorders, it is a measure of the common mental health problems/domains of depression, anxiety, somatic symptoms and social withdrawal. Of the various versions (60, 30, 28 and 12 items), the 28-item version is the one generally used. The GHQ has four subscales: somatic symptoms (GHQ–A), anxiety and insomnia (GHQ–B), social dysregulation (GHQ–C) and severe depression (GHQ–D), with each subscale represented by seven questions. The total possible score on the GHQ-28 ranges from 0 to 84 and allows for means and distributions to be calculated for both the global total and the four subscales. Using the alternative binary scoring method, with the two least symptomatic answers scoring 0 and the two most symptomatic answers scoring 1, the 28-item version classifies any score exceeding the threshold value of 4 as achieving “psychiatric caseness” [14]. The GHQ-28 has been found to perform well and be remarkably robust [23] with reliability coefficients ranging from 0.78 to 0.95 in various studies [24]. In our study, the subjects self-administered a GHQ-28 questionnaire every day just before the evening collection of saliva. Individual subscales are used for providing individual diagnostic or profile information.

### Saliva collection

Sampling kits consisting of a polypropylene cup (14 × 17 × 40.3 mm^3^) and a collection swab (sterilized dental cotton, 8 × 12.5 mm^2^) were used to correct the saliva samples. Each subject received three sets of the saliva sampling kit. Saliva samples were collected at three time points: 7:00 am (morning, M), 12:00 pm (noon, N) and 5:00 pm (early evening, E). Subjects were asked to refrain from from drinking liquids and from eating candy or gum for at least 1 h before the sample collection. To collect the saliva sample, the swab was placed under the tongue for 3 min, allowed to saturate with pooled saliva and then placed in the cup. The cups were stored in the freezer compartment of the home refrigerator and all kits returned to the school collection on 17 July. Here, the swabs were squeezed using a medical syringe and the expressed saliva stored at −80 °C until analysis.

### Salivary cortisol analysis

The collected saliva samples were batch processed by centrifugation at 1,789 G (100 mm radius and 4,000 rpm) for 5 min, and the resulting supernatant was stored at 4 °C using a cooling centrifuge (Himac CF15R, Hitachi Koki Co., Ltd.). Concentrations of salivary cortisol were analyzed using cortisol enzyme-linked immunosorbent assay kits (1-3002: Salimetrics LLC, PA) and a plate reader (450 nm measurement wavelength; ARVO MX; Perkin Elmer Life Science, Boston, MA).

### Data analytic strategy

All analyses were performed with the Statistical Package for the Social Sciences (SPSS), version 20.0 (SPSS Inc, Chicago, IL). Based on the GHQ-D subscale score, the subjects were divided into two groups: those with a score of 0 were assigned to the “low” symptoms, and those scoring 1 and above on any of the assessments were assigned to the “high” symptom group. Within-group comparisons were performed using the Wilcoxon test. Two-group comparisons were performed using the Mann-Whitney test. A *p* value < 0.05 was taken to represent statistical significance. Unless otherwise stated, all of the data are expressed as the mean ± SD. Receiver-operating characteristic (ROC) curves were generated to verify the discriminative ability of the salivary cortisol levels to detect subjects assigned to the “high” group for the GHQ-D subscale; areas under these curves (AUC) were calculated to provide an overall summary of the diagnostic accuracy of the salivary cortisol levels with the diagnostic ability classified into three levels: poor when 0.50 ≤ AUC < 0.69, good when 0.70 ≤ AUC < 0.89 and excellent when 0.90 ≤ AUC < 1.

## Results

### Sample characteristics

In total, 63 healthy students (37 boys and 26 girls; mean age, 14.29 ± 0.51 years) were initially enrolled in this study. There were no dropouts. Thus, the final cohort used for analyses included 63 subjects.

### Psychological assessment for depression

**Fig. 2:**
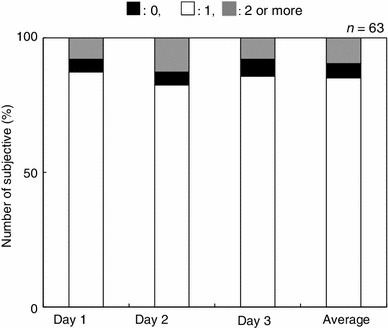
Distribution the GHQ-28 depression subscale. Scores across the three assessment days

Figure 2 summarizes the distribution of the scores for the GHQ-D subscale for depression. From the cohort of 63 subjects, 49 (78 %) were classified into the low symptom group, and the remaining 14 subjects (22 %) were classified into the high symptom group.

### Salivary cortisol

**Fig. 3:**
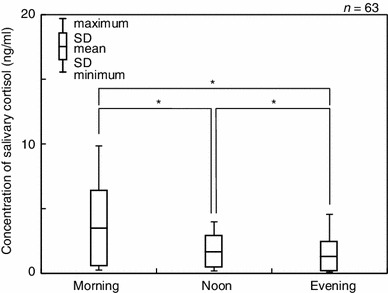
Temporal changes in the concentrations of salivary cortisol. *p < 0.05

Concentrations of salivary cortisol ranged between 0.10 and 9.86 ng/ml with a mean value of 2.01 ± 1.67 ng/ml. The mean salivary cortisol levels at each of the daily sampling time points were 3.41 ± 2.02 (M), 1.55 ± 0.83 (N) and 1.06 ± 0.79 ng/ml (E), respectively. The salivary cortisol levels decreased significantly over the course of the day (Fig. 3). As shown in Table 1, morning samplings showed that the salivary cortisol levels differed significantly by gender with boys showing lower levels (2.82 ± 1.54 ng/ml) than girls (4.24 ± 1.54 ng/ml). In contrast, the gender differences in salivary cortisol levels were less distinct in the evening samples with 1.03 ± 0.51 ng/ml for boys and 1.10 ± 0.53 ng/ml for girls, respectively.

**Table 1:** Salivary cortisol concentrations (ng/ml, mean ± SD) by gender and GHQ depression subscores (GHQ-D)

Group	*n*	Morning		Noon		Evening	
Male	37	2.82	1.54	1.36	0.42	1.03	0.51
Female	26	4.24	1.54	1.82	0.94	1.10	0.53
Low group total	49	3.33	1.77	1.61	0.82	1.14	0.79
Male	32	2.97	1.27	1.46	0.88	1.12	0.75
Female	17	3.99	2.29	1.89	0.58	1.16	0.67
High group total	14	3.71	2.84	1.37	0.86	0.79	0.64
Male	5	1.92	1.03	0.77	0.25	0.46	0.12
Female	9	4.72	1.58	1.70	0.83	0.97	0.68
Total	63	3.41	2.02	1.55	0.83	1.06	0.79

**Fig. 4:**
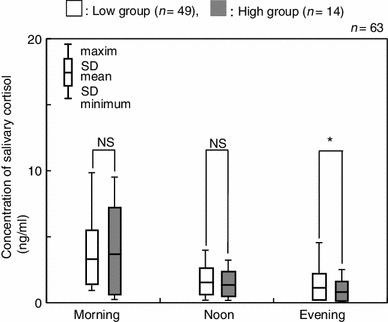
Comparison of salivary cortisol concentrations between high and low groups at each time point. *p < 0.05, *NS* not significant

**Fig. 5:**
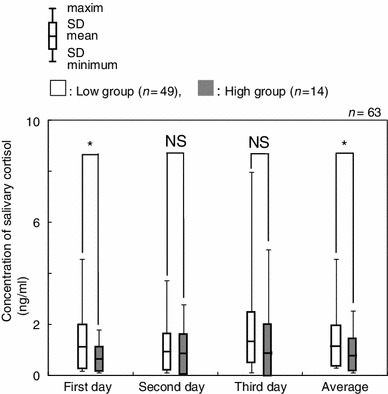
Comparison between high and low groups by the evening time point during this experiment. *p < 0.05, *NS* not significant

Salivary cortisol levels were compared between the two groups using the average concentration across corresponding time points (Fig. 4). The salivary cortisol levels in the low group were 3.33 ± 1.77 ng/ml (M), 1.61 ± 0.82 ng/ml (N) and 1.14 ± 0.79 ng/ml (E), respectively. The salivary cortisol levels in the high group were 3.71 ± 2.84 ng/ml (M), 1.37 ± 0.86 ng/ml (N) and 0.79 ± 0.64 ng/ml (E), respectively. A significant difference was observed between the salivary cortisol levels of the two groups for the average of the evening measurements (Fig. 4, *p* < 0.05). No significant differences were observed between the two groups for the morning (M) or afternoon (N) time points. On further examination of the evening time point (E), the low group showed statistically higher salivary cortisol levels than that of the high group on the first day and the total average (Fig. 5, *p* < 0.05).

**Fig. 6:**
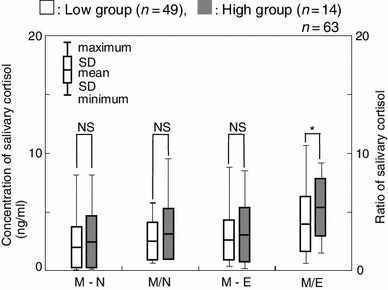
Comparison of salivary cortisol concentrations between high and low groups by the difference (M–N and M–E) and slope (M/N and M/E) in salivary cortisol. * *p* < 0.05, *NS* not significant

**Fig. 7:**
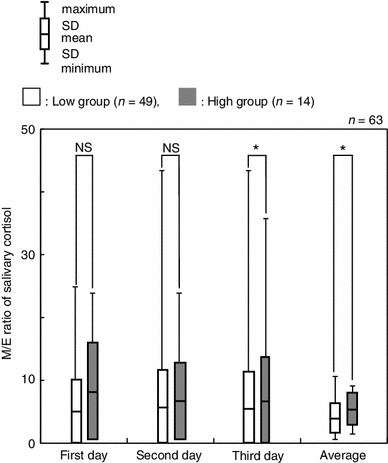
Comparison between high and low groups by the M/E ratio during this experiment. *p < 0.05, *NS* not significant

To examine whether there were any differences in the magnitude of the daily change, salivary cortisol levels were then compared in terms of the difference and the slope. A significant difference was observed between the two groups in terms of M/E ratio when the data collected over 3 days were used (*p* < 0.05, Fig. 6). In this case, the M/E ratios of the low and high groups were 3.86 ± 2.37 and 5.37 ± 2.50, respectively. On further examination of the M/E ratio, the high group showed statistically higher salivary cortisol levels than those of the low groups on the 3rd day and the total average (*p* < 0.05, Fig. 7).

On the other hand, when the subjects were divided into two groups by the score of GHQ-A (somatic symptoms), GHQ-B (anxiety and insomnia) or GHQ-C (social dysregulation), there were no significant differences between the two groups in the time points, difference and slope.

### Validation of diagnostic performance by ROC curve

**Fig. 8:**
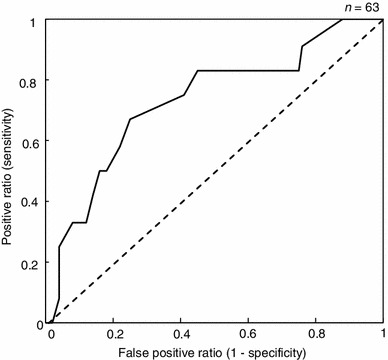
ROC curve using the M/E ratio of the concentration of salivary cortisol over 3 days

The AUC was calculated to be 0.5 when one time point, such as M, N or E, was considered. In the evening data point, the AUC of the first day and total average were 0.71 and 0.66, respectively (Table 2). However, the AUC was reached at 0.75 when the M/E ratio and the total average were used (Fig. 8). The diagnostic performance was classified as good.

**Table 2:** Calculated results of AUC for each condition

Data of salivary cortisol	Time point	AUC
Evening (E)	First day	0.71
	Total average	0.66
M/E ratio	Third day	0.35
	Total average	0.75

## Discussion

Under the GHQ-28 subscale used to assess severe depression, 22 % of the adolescent subjects classified into the high symptom group (Fig. 2). A previous study utilizing GHQ-28 reported that 9–18 % of subjects without a prior traumatic experience manifest depression [23]. Other reports indicated that the rates of depression increased to 40–44 % if the subjects attempted suicide or suffered from flooding [25, 26]. In our study, the rates of depression were intermediate between these two results. We believe that the lower than anticipated rates of depression in the group impacted by the Tohoku earthquake and accompanying tsunami can be explained by the fact that for many the state of depression may have ameliorated over the 1 year that had passed since that traumatic event.

The mean salivary cortisol levels for the morning, afternoon and evening collections (3.41, 1.55 and 1.06 ng/ml) in our study corresponded to ranges reported previously by other studies: 3.43–10.12 ng/ml in the morning, 2.07–4.01 ng/ml in the afternoon and 2.13–7.09 ng/ml in the evening (Fig. 3) [27–29]. Thus, our findings validated the circadian levels of salivary cortisol reported by other researchers.

Based on the GHQ-D subscores, the subjects could be clustered into low and high symptom groups. By themselves, the patterns of diurnal changes in the salivary cortisol concentration did not significantly differ between the two groups. Thus, our findings suggest that it will be difficult distinguish individuals with depressive symptoms on the basis of point measurements of salivary cortisol. However, when the two groups were compared using the variations in salivary cortisol over the course of 3 consecutive days, the high symptom group showed significantly higher morning/evening ratios in their salivary cortisol levels compared with the low group. Accordingly, we believe that the assessment of salivary cortisol levels over multiple days is more indicative of depressive states in adolescents. Analyzed by means of receiver-operating characteristic (ROC) curves, the M/E ratio of the salivary cortisol had good discriminating power (0.75) for discriminating between subjects with and without depressive symptoms measured by the GHQ-28 (Fig. 8). Our study indicates that the state of depressive symptoms in adolescents can be assessed by salivary cortisol levels measured twice a day, in the morning and in the evening, over 3 consecutive days.

## Conclusions

A significant subset (22 %) of adolescents in the area affected by the Great East Japan Earthquake were classified as having high depressive symptoms even 1 year after the event. We explored the optimal timing and frequency of the salivary cortisol measurement in order to assess depressive symptoms. Both the evening time point and morning/evening ratios of salivary cortisol levels were useful in discriminating between subjects with and without depression. ROC curve analysis indicates that repeated measurements of salivary cortisol over 3 consecutive days can identify adolescents with depressive symptoms and point to the potential utility of salivary cortisol in exploring the neurobiological underpinnings of stress disorders such as depression. Technology to enable point of use measurement of salivary cortisol would help clarify the neurobiological underpinnings of stress disorders such as depression.

## References

[1] Mimura N, Yasuhara K, Kawagoe S, Yokoki H, Kazama S (2011). Damage from the great East Japan earthquake and tsunami—a quick report. Mitig Adapt Strateg Glob Change.

[2] Hayashi K, Tomita N (2012). Lessons learned from the great East Japan earthquake impact on child and adolescent health. Asia Pac J Public Health.

[3] Kyutoku Y, Tada R, Umeyama T, Harada K, Kikuchi S, Watanabe E, Liegey-Dougall A, Dan I (2012). Cognitive and psychological reactions of the general population 3 months after the 2011 Tohoku earthquake and tsunami. PLoS One.

[4] Connor KM, Foa EB, Davidson JRT (2006). Practical assessment and evaluation of mental health problems following a mass disaster. J Clin Psychiatry.

[5] Goenjian AK, Pynoos RS, Steinberg AM, Najarian LM, Asarnow JR, Karayan I, Ghurabi M, Fairbanks LA (1995). Psychiatric comorbidity in children after the 1988: earthquake in Armenia. J Am Acad Child Adolesc Psychiatry.

[6] Vogel JM, Vernberg EM (1993). Part 1: children’s psychological responses to disasters. J Clin Child Psychol.

[7] Thienkrua W, Cardozo B, Chakkraband M, Guadamuz TE, Pengjuntr W, Tantipiwatanaskul P, Sakornsatian S, Ekassawin S, Panyayong B, Varangrat A, Tappero JW, Schreiber M, van Griensven F, Thailand Post-Tsunami Mental Health Study Group (2006). Symptoms of posttraumatic stress disorder and depression among children in tsunami-affected areas in southern Thailand. JAMA.

[8] Rickwood DJ, Deane FP, Wilson CJ (2007). When and how do young people seek professional help for mental health problems?. Med J Aust.

[9] Nemeroff R, Levitt JM, Faul L, Wonpat-Borja A, Bufferd S, Setterberg S, Jensen PS (2008). Establishing ongoing, early identification programs for mental health problems in our schools: a feasibility study. J Am Acad Child Adolesc Psychiatry.

[10] Cuijpers P, van Straten A, Smits N, Smit F (2006). Screening and early psychological intervention for depression in schools. Eur Child Adolesc Psychiatry.

[11] Shaffer D, Scott M, Wilcox H, Maslow C, Hicks R, Lucas CP, Garfinkel R, Greenwald S (2004). The Columbia suicide screen: validity and reliability of a screen for youth suicide and depression. J Am Acad Child Adolesc Psychiatry.

[12] Brewin CR (2005). Systematic review of screening instruments for adults at risk of PTSD. J Trauma Stress.

[13] Brewin CR, Rose S, Andrews B (2002). Brief screening instrument for post-traumatic stress disorder. Br J Psychiatry.

[14] Jackson C (2007). The general health questionnaire. Occup Med.

[15] Marsella AJ, Friedman MJ, Gerrity ET, Scurfield RM (1996). Ethnocultural aspects of post traumatic stress disorder: issues, research, and clinical applications.

[16] Triffleman EG, Pole N (2010). Future directions in studies of trauma among ethnoracial and sexual minority samples: commentary. J Consult Clin Psychol.

[17] Borkovec T, Castonguay L, Newman M (1997). Measuring treatment outcome for posttraumatic stress disorder and social phobia: a review of current instruments and recommendations for future research. Measuring patient changes: in mood, anxiety, and personality disorders.

[18] Shetty V, Yamaguchi M (2007). Salivary biosensors for screening trauma-related psychopathology. Oral Maxillofac Surg Clin N Am.

[19] Aardal-Eriksson E, Eriksson TE, Thorell L-H (2001). Salivary cortisol, posttraumatic stress symptoms, and general health in the acute phase and during 9-month follow-up. Biol Psychiatry.

[20] Hellhammer DH, Wüst S, Kudielka BM (2009). Salivary cortisol as a biomarker in stress research. Psychoneuroendocrinology.

[21] Yamaguchi M, Matsuda Y, Sasaki S, Sasaki M, Kodama Y, Imai Y, Niwa D, Shetty V (2013). Immunosensor with fluid control mechanism for salivary cortisol analysis. Biosens Bioelectron.

[22] Goldberg DP, Gater R, Sartorius N (1997). The validity of two versions of the GHQ in the WHO study of mental illness in general health care. Psychol Med.

[23] Sweeting H, Young R, West P (2008). GHQ increases among Scottish 15 year old 1987–2006. Soc Psychiatry Psychiatr Epidemiol.

[24] Jackson C (2007). The general health questionnaire. Occup Med.

[25] Sweeting H, Young R, West P (2008). GHQ increases among Scottish 15 year old 1987–2006. Soc Psychiatry Psychiatr Epidemiol.

[26] Sawitri A, Sa-nguansri T, Edwards SJ (2004). The flooding of Hat Yai: predictors of adverse emotional responses to a natural disaster. Stress Health.

[27] Schommer NC, Kudielka BM, Hellhammer DH, Kirschbaum C (1999). No evidence for a close relationship between personality traits and circadian cortisol rhythm or a single cortisol stress response. Psychol Rep.

[28] Heiser P, Dickhaus B, Schreiber W, Clement HW, Hasse C, Hennig J, Remschmidt H, Krieg JC, Wesemann W, Opper C (2000). White blood cells and cortisol after sleep deprivation and recovery sheep in human. Eur Arch Psychiatr Clin Neurosci.

[29] Tchiteya BM, Lecours AR, Elie R, Lupien SJ (2003). Impact of a unilateral brain lesion on cortisol section and emotional state: anterior/posterior dissociation in humans. Psychoneuroendocrinology.

